# Circumsporozoite Protein-Specific K^d^-Restricted CD8+ T Cells Mediate Protective Antimalaria Immunity in Sporozoite-Immunized MHC-I-K^d^ Transgenic Mice

**DOI:** 10.1155/2014/728939

**Published:** 2014-07-15

**Authors:** Jing Huang, Tiffany Tsao, Min Zhang, Moriya Tsuji

**Affiliations:** ^1^HIV and Malaria Vaccine Program, Aaron Diamond AIDS Research Center, Affiliate of The Rockefeller University, 455 First Avenue, New York, NY 10016, USA; ^2^Department of Pathology, New York University School of Medicine, 545 First Avenue, New York, NY 10016, USA

## Abstract

Although the roles of CD8+ T cells and a major preerythrocytic antigen, the circumsporozoite (CS) protein, in contributing protective antimalaria immunity induced by radiation-attenuated sporozoites, have been shown by a number of studies, the extent to which these players contribute to antimalaria immunity is still unknown. To address this question, we have generated C57BL/6 (B6) transgenic (Tg) mice, expressing K^d^ molecules under the MHC-I promoter, called MHC-I-K^d^-Tg mice. In this study, we first determined that a single immunizing dose of IrPySpz induced a significant level of antimalaria protective immunity in MHC-I-K^d^-Tg mice but not in B6 mice. Then, by depleting various T-cell subsets *in vivo*, we determined that CD8+ T cells are the main mediator of the protective immunity induced by IrPySpz. Furthermore, when we immunized (MHC-I-K^d^-Tg × CS-Tg) F1 mice with IrPySpz after crossing MHC-I-K^d^-Tg mice with PyCS-transgenic mice (CS-Tg), which are unable to mount PyCS-specific immunity, we found that IrPySpz immunization failed to induce protective antimalaria immunity in (MHC-I-K^d^-Tg × CS-Tg) F1 mice, thus indicating the absence of PyCS antigen-dependent immunity in these mice. These results indicate that protective antimalaria immunity induced by IrPySpz in MHC-I-K^d^-Tg mice is mediated by CS protein-specific, K^d^-restricted CD8+ T cells.

## 1. Introduction

Malaria is a severe disease that ranks among the most prevalent infections in tropical areas throughout the world. Approximately 250–300 million people become infected yearly with relatively high rates of morbidity and mortality. The WHO estimates that every year nearly one million children die of malaria in Africa alone [1]. The widespread occurrence and the increasing incidence of malaria in many countries, caused by drug resistant parasites and insecticide resistant vectors (*Anopheles* mosquitoes), underscore the need for developing new methods of controlling this disease, which include more effective vaccines.

Most vaccine efforts are directed against the preerythrocytic stages (sporozoites (Spz) and liver stages) and blood stages [2]. The finding that vaccination with radiation-attenuated sporozoites (IrSpz) can induce complete protection (i.e., sterile immunity) against malaria infection not only in experimental animals but also in man [3–7] demonstrated the feasibility of effective vaccination against this disease. A number of mouse studies to date using* Plasmodium yoelii* and* P. berghei* parasites for challenge have shown that protective immunity against preerythrocytic stages is mediated in part by T cells, particularly CD8+ T cells. Firstly, the major role for CD8+ T cells was shown by studies in which* in vivo* depletion of CD8+ T cells abrogated Spz-induced protective immunity in mice [8, 9]. Secondly, the adoptive transfer of CD8+ T-cell clones specific for the immunodominant CD8+ T-cell epitope, SYVPSAEQI, of the* P. yoelii* circumsporozoite (PyCS) protein, a major Spz antigen, confers protection against Spz challenge in naïve mice [10, 11]. More recently, by using transgenic (Tg) mice expressing a T-cell receptor (TCR), based on the TCR sequence of CD8+ T cells recognizing the SYVPSAEQI epitope, transgenic CD8+ T cells were shown to mediate protection against malaria [12]. Finally, Hoffman's group has recently shown that intravenous (IV) immunization of IrSpz vaccine is very effective in inducing a high frequency of malaria-specific CD8+ T cells in the liver of nonhuman primates and mice and, furthermore, conferring protection in mice [13]. More recently the same group showed that immunization of multiple doses of their IrPfSPZ vaccine by IV conferred protection in six out of six vaccines against malaria challenge [14].

Thus, a number of studies have shown that CD8+ T cells can significantly contribute to the protective immunity against the liver stages of malaria parasites in mice [8–16]. However, it is still largely unknown to which extend CD8+ T cells, particularly those specific for the CS protein, can mediate the protection induced by IrSpz. To address this key question, we have taken a novel approach. C57BL/6 (B6) mice express MHC-class-I molecule, H-2K^b^ (K^b^), but lack H-2K^d^ (K^d^) molecule, whereas BALB/c mice express the K^d^ molecules. We have generated transgenic (Tg) B6 mice that express the K^d^ molecules on all nucleated cells under the major histocompatibility complex- (MHC-) I promoter, which we call MHC-I-K^d^-Tg mice [17]. As described above, the immunodominant T-cell epitope of the PyCS protein, SYVPSAEQI, is presented by H-2K^d^ molecules to CD8+ T cells and is known to be the only epitope that can induce protective CD8+ T cells against malaria [10, 11], underscoring the importance of generating MHC-I-K^d^-Tg mice. These MHC-I-K^d^-Tg mice were used to further refine the role of CD8+ T cells in protective antimalaria immunity induced by IrPySpz. Furthermore, by crossing our MHC-I-K^d^-Tg mice with PyCS-transgenic mice (CS-Tg), in which mice are unable to induce PyCS-specific immunity [18], we have generated (MHC-I-K^d^-Tg × CS-Tg) F1 mice and used it to study the role of CS antigen in mediating protective antimalaria immunity induced in IrPySpz-immunized MHC-I-K^d^-Tg mice.

## 2. Materials and Methods

### 2.1. Mice

B6 mice and BALB/c mice were purchased from the Jackson Laboratory (Bar Harbor, ME). Transgenic mice (MHC-I-K^d^) expressing H-2K^d^ shared allele by BALB/c mice under the control of the H-2K^b^ promoter on the B6 background were derived as previously described [17]. CS transgenic mice expressing CS gene of* P. yoelii* (17X NL) on the B6 background, CS-Tg mice [18], were kindly provided to us by Dr. Victor Nussenzweig at New York University. (MHC-I-K^d^-Tg × CS-Tg) F1 mice were generated by crossing CS-Tg mice to MHC-I-K^d^-Tg mice.

### 2.2. Antibodies

The following monoclonal antibodies (mAb) were purchased from BioLegend (San Diego, CA) and used for a flow cytometric analysis: purified anti-CD16/32 (clone 93), Alexa Fluor 647-labeled anti-H-2K^d^ (clone SF1-1.1), Pacific Blue-labeled anti-F4/80 (clone BM8), PE-Cy7-labeled anti-CD11b (clone M1/70), PerCP-Cy5.5-labeled anti-I-A^b^ (clone AF6-120.1), PE-labeled anti-CD11c (clone N418), FITC-labeled anti CD3 (17A2), APC-labeledanti-CD4 (clone RM4-5), and Pacific Blue-labeled anti-CD8 (clone 53-6.7).

### 2.3. Flow Cytometric Analysis

Murine cells were incubated with unlabeled anti-CD16/CD32 mAb for 10 min at RT and later incubated with the respective mAbs described in the preceding section. All cells were costained with propidium iodide (Sigma-Aldrich, St. Louis, MO) to exclude nonviable cells. Flow cytometric data collection was performed using an LSR II Flow Cytometer (BD Biosciences, San Jose, CA). Subsequent data analyses were performed using FlowJo software (Tree Star Inc.).

### 2.4. Parasites and Immunization

Female* Anopheles stephensi* mosquitoes infected with* P. yoelii* 17×NL strain were purchased from the New York University insectary.* P. yoelii* sporozoites were isolated from the salivary glands of infected* A. stephensi* mosquitoes 14 days after the mosquitoes had received an infectious blood meal. Sporozoites for immunization were attenuated after giving 15,000 rads by a gamma-irradiator. Mice were immunized with 5 × 10^4^ to 1 × 10^5^ irradiated sporozoites suspended in RPMI with 2% mouse sera by IV or intramuscular (IM) injection.

### 2.5. Depletion of T-Cell Subsets* In Vivo*

MHC-I-K^d^-Tg mice were intraperitoneally injected with 250 *μ*g or 500 *μ*g of rat mAbs against CD4 (clone GK 1.5) or CD8 (clone YTS 169), respectively, on day −3 and day −1 prior to challenge with live* P. yoelii* sporozoites. The status of* in vivo* depletion of the corresponding T-cell subsets was assessed by flow cytometric assay, using anti-CD4 (clone RM4-5) and anti-CD8 (clone 53-6.7) mAbs.

### 2.6. Sporozoite Challenge and Assessment of Parasite Burden in the Liver

Mice were challenged by IV injection of viable* P. yoelii* sporozoites with varied doses from 1 × 10^4^ to 5 × 10^4^ per mouse. Parasite burden in the liver was determined 42 h after the challenge by measuring parasite-specific 18S rRNA using a quantitative real-time reverse transcription-PCR method with the 7500 Fast Real-Time PCR System (Applied Biosystems). Parasite burden was described as a ratio of the absolute copy number of parasite-specific 18S rRNA to that of mouse GAPDH.

### 2.7. Statistics

All of the statistical analyses were done using GraphPad Prism (version 5.03) (GraphPad Software Inc.). In the challenge experiment, parasite load in the liver was determined by a real-time RT-PCR. The values were then log-transformed and analyzed by 1-way ANOVA, followed by Dunnett's test. *P* < 0.01 is considered statistically significant.

## 3. Results and Discussion

**Figure 1:**
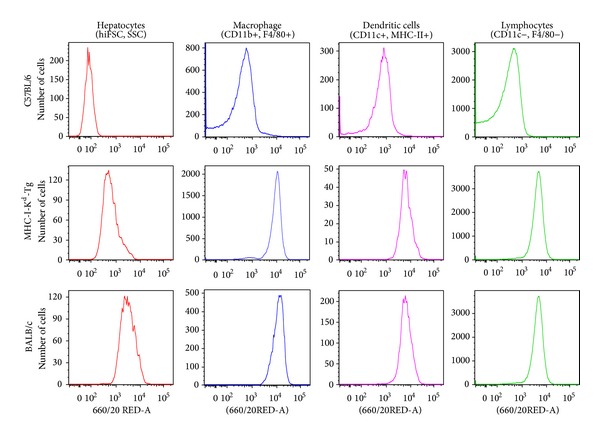
*Phenotype of MHC-I-K* ^*d*^ *-Tg mice*. Histograms show H-2K^d^ expression levels on hepatocytes, macrophages, dendritic cells, and lymphocytes of C57BL/6 (B6), MHC-I-K^d^-Tg, and BALB/c. Hepatocytes were isolated from mouse liver and then gated based on high FSC/SSC parameters. Macrophages were gated on CD11b+/F4/80+ and CD11c+/MHC II+ were used to define dendritic cells. Lymphocytes were identified by CD11c−/F4/80−.

MHC-I-K^d^-Tg mice that express BALB/c mouse-derived H-2K^d^ allele under the control of the H-2K^b^ promoter on the B6 background were established previously in our laboratory [17]. The K^d^ expression profile of MHC-I-K^d^-Tg mice was extensively investigated by flow cytometric analysis in various cells including hepatocytes, macrophages, dendritic cells, and lymphocytes as shown in Figure 1. B6 mice and BALB/c mice were used as K^d^ negative control and K^d^ positive control, respectively. As shown in Figure 1, K^d^ expression level on hepatocytes of MHC-I-K^d^-Tg mice was only slightly lower than that of BALB/c mice but still significantly higher than that of B6 mice lacking K^d^ expression. In Figure 1, MHC-I-K^d^-Tg mice showed similar expression levels of K^d^ on macrophages, dendritic cells, and lymphocytes as that on the corresponding cells of BALB/c mice. This suggests that, as expected, the MHC-I-K^d^-Tg mice express K^d^ to the extent very similar to that of BALB/c mice, even with B6 mice background.

**Figure 2:**
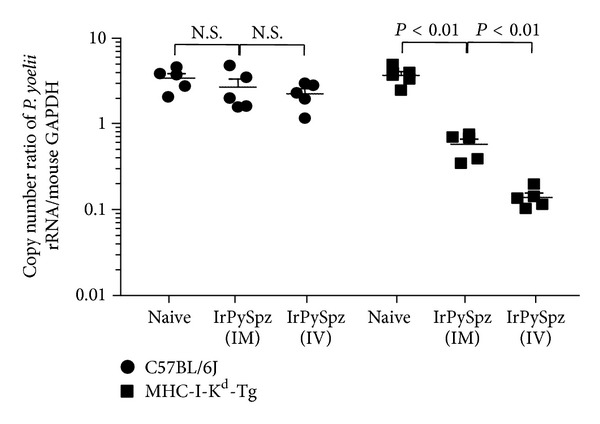
*Protection against malaria infection after a single immunizing dose of IrPySpz*. Groups of naive B6 mice and MHC-I-K^d^-Tg mice (5 per group) were immunized with 1 × 10^5^ IrPySpz via IV or IM injection. Protection assay was performed by challenge with 5 × 10^4^ viable* P. yoelii* sporozoites via IV injection 2 weeks after immunization. Naïve B6 mice and MHC-I-K^d^-Tg mice were also challenged as infection control. Parasite burden was described as a ratio of the absolute copy number of parasite-specific 18s rRNA to that of mouse GAPDH. *P* < 0.01 is considered statistically significant, whereas N.S. means “not significant” in Figures 2–4.

The protection between MHC-I-K^d^-Tg mice and B6 mice against challenge of infectious* P. yoelii* sporozoites was evaluated after immunization with radiation-attenuated* P. yoelii* sporozoites (IrPySpz) by route of either IV or IM injections. As shown in Figure 2, IrPySpz immunization resulted in a statistically significant reduction (*P* < 0.01) in parasite load via both IV and IM injections in the livers of MHC-I-K^d^-Tg mice. There was no such reduction in those of B6 mice in either IV or IM IrPySpz immunization routes. This is presumably because B6 mice lack K^d^ molecules that can present PyCS-derived CD8+ T-cell epitope for the induction of protective antimalarial CD8+ T cells. The challenge results in Figure 2 also showed that IrPySpz immunization in MHC-I-K^d^-Tg mice via IV injection provided significantly more protection (*P* < 0.01) by way of liver stage parasite load reduction than the same immunization via IM injection. This finding corroborates the finding recently observed in humans showing that vaccination by IV of irradiated* P. falciparum* sporozoites induced protection [14].

**Figure 3:**
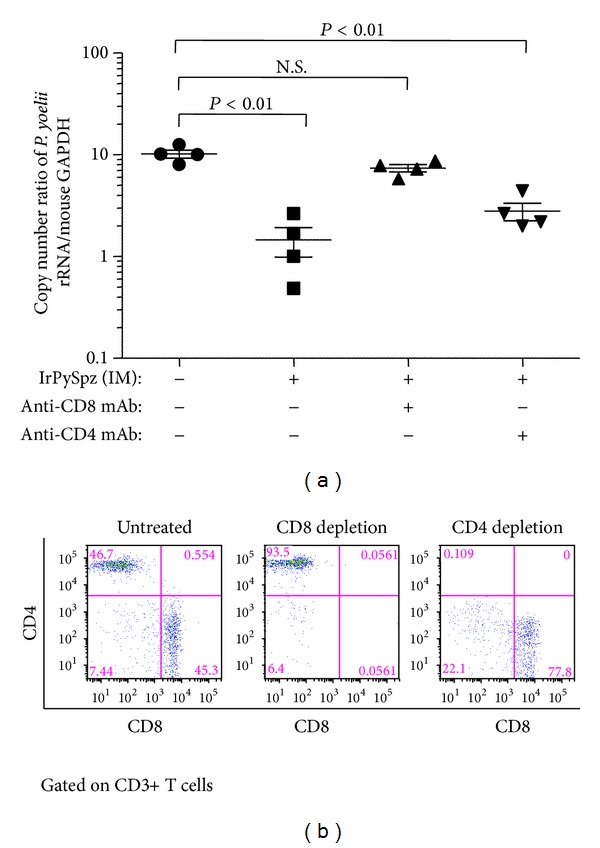
*Protection against plasmodium infection in IrPySpz-immunized MHC-I-K* ^*d*^ *-Tg mice after depletion of CD8+ T cells or CD4+ T cells.* (a) Groups of MHC-I-K^d^-Tg mice (4 per group) were immunized with 1 × 10^5^ IrPySpz via IM injection following challenge with 5 × 10^4^ viable* P. yoelii* sporozoites via IV injection at 2 weeks after immunization. Some cohorts of mice were treated with rat anti-CD4 mAb (GK1.5) or anti-CD8 mAb (YTS 169) 3 days and 1 day prior to challenge with* P. yoelii* sporozoites. Naive MHC-I-K^d^-Tg mice were challenged as infection control. The value of parasite burden was described previously. (b) The dot plots by flow cytometric analysis represent the frequency of total CD4+ and CD8+ T cells among the CD3+ T-cell population of splenocytes, following the indicated antibody treatment to IrPySpz-immunized MHC-I-K^d^-Tg mice.

In order to investigate which type of lymphocytes mediates the protective immunity against preerythrocytic stages of malaria, we depleted either CD4+ T cells or CD8+ T cells from MHC-K^d^-Tg mice immunized IM with a single dose of IrPySpz. As shown in Figure 3(a), depletion of CD8+ T cells remarkably abolished the inhibition of the liver stage development in IrPySpz-immunized MHC-I-K^d^-Tg mice followed by live PySpz challenge. The levels of parasite load in CD8+ T-cell-depleted, IrPySpz-immunized mice were very similar to that of unimmunized naïve mice, following infectious PySpz challenge. The depletion of CD4+ T cells, meanwhile, significantly diminished but failed to abolish the inhibition observed in IrPySpz-immunized, PySpz-challenged MHC-I-K^d^-Tg mice (Figure 3(a)).  Figure 3(b) shows that the* in vivo* administration of monoclonal antibody against CD4+ or CD8+ T cells efficiently depleted each respective T-cell population. This finding corroborates a previous study, in which BALB/c mice carrying H-2d haplotype were immunized intravenously with IrPySpz [9], and strongly suggests that IrPySpz-induced antimalaria protection observed in MHC-I-K^d^-Tg mice is largely dependent on CD8+ T cells but not on CD4+ T cells. CD8+ T-cell-dependent protection observed in MHC-I-K^d^-Tg mice would make sense in view of the presence of K^d^ molecules in MHC-I-K^d^-Tg mice, which should be able to present an immunodominant CD8+ T-cell epitope, SYVPSAEQI, derived from the PyCS protein, thus eliciting potent and protective CD8+ T-cell response against malaria.

**Figure 4:**
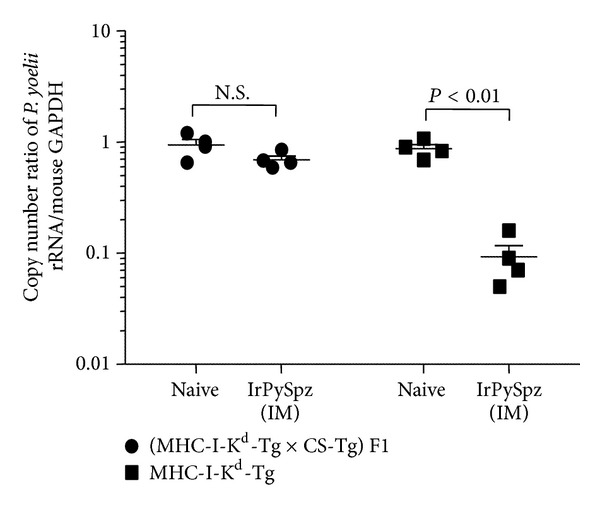
*Protection against plasmodium infection in IrPySpz-immunized MHC-I-K* ^*d*^ *-Tg mice and (MHC-I-K* ^*d*^ *-Tg *×* CS-Tg) F1 mice*. (MHC-I-K^d^-Tg × CS-Tg) F1 mice were generated by crossing CS-Tg mice to MHC-I-K^d^-Tg mice. Groups (4 mice per group) of naive MHC-I-K^d^-Tg mice and MHC-I-K^d^-Tg × CS-Tg mice were immunized with 5 × 10^4^ IrPySpz via IM injection. Protection assay was performed by challenge with 1 × 10^4^ infectious* P. yoelii* sporozoites via IV injection 2 weeks after immunization. Naïve MHC-I-K^d^-Tg mice and (MHC-I-K^d^-Tg × CS-Tg) F1 mice were also challenged as infection controls. The value of parasite burden was described previously.

However, it is still unclear to which extent a single immunizing dose of IrPySpz would induce protective immunity mediated by PyCS antigen-specific CD8+ T-cell response. Although a whole sporozoite consists of more than one thousand antigens, CS protein is shown to be a dominant antigen that can mediate the protective immunity against preerythrocytic stages of malaria. This was verified by using PyCS antigen transgenic mice (CS-Tg mice) that were tolerant to CS-T-cell epitopes, as PyCS-specific CD8+ T-cell response was not detected in the CS-Tg mice with BALB/c background upon IrPySpz immunization [18]. Therefore, in order to determine the contribution of PyCS antigen in overall protective antimalaria immunity induced by IrPySpz, we decided to cross MHC-I-K^d^-Tg mice with CS-Tg mice and generated (MHC-I-K^d^-Tg × CS-Tg) F1 mice. Then we immunized (MHC-I-K^d^-Tg × CS-Tg) F1 mice, as well as MHC-I-K^d^-Tg mice, with a single dose of IrPySpz and compared the level of protective antimalaria immunity between the two groups of mice upon challenge with live PySpz.  Figure 4 shows that, in contrast to the significant level of protective antimalaria immunity observed in IrPySpz-immunized MHC-I-K^d^-Tg mice, a single immunizing dose of IrPySpz failed to mount a significant level of protective immunity in (MHC-I-K^d^-Tg × CS-Tg) F1 mice. These findings suggest that the protective antimalaria immunity induced in MHC-K^d^-Tg mice by a single immunizing dose of IrPySpz is dependent on the immunity against the PyCS antigen.

## 4. Conclusions

Using transgenic B6 mice expressing K^d^ molecules in all nucleated cells under MHC-class-I promoter, we investigated in the current studies the nature of protective antimalaria immunity induced by immunization with radiation-attenuated* P. yoelii* sporozoites, IrPySpz. Firstly, we found that a single immunizing dose of IrPySpz could induce a significant level of antimalaria protective immunity in MHC-I-K^d^-Tg mice, but not in B6 mice, likely due to the presence of K^d^ molecule. Then we determined that CD8+ T cells are the main mediators of the protective immunity induced by IrPySpz by depleting various T-cell subsets* in vivo* from IrPySpz-immunized MHC-I-K^d^-Tg mice. Furthermore, when we generated (MHC-I-K^d^-Tg × CS-Tg) F1 mice, by crossing the MHC-I-K^d^-Tg mice with PyCS-transgenic (CS-Tg) mice that fail to mount PyCS-specific immunity, and immunized them with IrPySpz, we found that IrPySpz failed to induce protective antimalaria immunity in (MHC-I-K^d^-Tg × CS-Tg) F1 mice, indicating that the protective immunity observed in MHC-I-K^d^-Tg mice depends on the immunity specific for the PyCS antigen. Altogether, in summary, our current studies indicate that IrPySpz-induced, protective antimalaria immunity in MHC-I-K^d^-Tg mice is dependent on CS protein-specific, K^d^-restricted CD8+ T cells.
